# Effects of a Mobile Educational Program for Colorectal Cancer Patients Undergoing the Enhanced Recovery After Surgery

**DOI:** 10.2174/1874434601812010142

**Published:** 2018-07-31

**Authors:** Bo-Yeoul Kim, Kyu-Joo Park, Seung-Bum Ryoo

**Affiliations:** 1 College of Nursing, Eulji University, Daejeon, Korea; 2 Department of Surgery, Seoul National University College of Medicine, Seoul National University Hospital, Seoul, Korea

**Keywords:** Anxiety, Colorectal cancer, Depression, Education, Quality of life, Self-efficacy, Depression

## Abstract

**Background:**

The Enhanced Recovery After Surgery (ERAS) program hastens recovery from colorectal cancer by shortening the treatment period and enabling a return to normal activities. However, patients with colorectal cancer treated under the ERAS program have fewer opportunities to consult with medical staff and receive education regarding self-care and experience more affective stress and anxiety.

**Objective:**

This study aimed to develop and assess an educational program for patients with colorectal cancer treated under the ERAS program, considering affective aspects.

**Method:**

Patients with colorectal cancer (*n* = 118) who underwent open colon surgery under the ERAS program were assigned alternately in the order of admission on a 1:1 basis to a treatment group (*n* = 59) and conventional care group (*n* = 59). The treatment group received a two-week mobile-based intervention, whereas the control group received conventional care. Quality of life, self-efficacy, anxiety, and depression were compared between the two groups.

**Results:**

The mobile web-based educational program significantly reduced the negative impact of surgery on the quality of life in the treatment group, compared with the conventional care group, and triggered a noticeable decline in anxiety and depression and increase in self-efficacy.

**Conclusion:**

The developed mobile web-based educational program effectively enhanced self-efficacy, positively impacted the quality of life, and reduced anxiety and depression. The program could have a positive effect on the quality of life of patients with colorectal cancer treated under the ERAS program.

## 1 INTRODUCTION

Colorectal cancer is one of the most common cancers, affecting approximately one million people around the world each year since 2010 and accounting for about 500,000 deaths annually [1]. With the world’s aging population, the incidence rate of colorectal cancer is increasing. Furthermore, with a survival rate of 87%, which is high compared to those of other serious cancers [2], patients with colorectal cancer often live with the illness for a longer period, similar to patients with other chronic diseases.

Most patients with colorectal cancer undergo surgery [3] and are likely to experience changes during the initial post-surgery stage, including pain (caused by the operational wound), fatigue, and changes in diet, mobilization, and bowel habits, as well as stress owing to concerns regarding family cancer history. Moreover, after surgery, patients are at risk of both physical and psychological distress, such as anxiety and depression. Therefore, the completion of active treatment requires continuing psychological care and support from others [4]. The transition from active treatment in a hospital to post-treatment care is critical to long-term health [5], and it is important that the distress is managed properly [6].

Since Kehlet and Wilmore [7] introduced the Enhanced Recovery After Surgery (ERAS) program, ERAS protocols have been shown to be useful for elective colorectal surgery [8, 9] and are being increasingly adopted to hasten recovery from such surgeries. To return to regular activities, ERAS patients require more care and support than those who go through the traditional process. Owing to their shorter hospital stay, patients treated under the ERAS program have less time to consult with medical staff; therefore, they receive less education and support regarding post-discharge care. Thus, a standardized care protocol to empower patients’ self-care abilities was developed and applied widely [10]; however, psychological problems such as affective stress, anxiety, and depression experienced by patients with colorectal cancer throughout diagnosis, surgery, hospital stay, and post-discharge have not been given due importance or appropriately explored. The transitional phase from hospital treatment to self-care at home is when patients most require intervention and feel insecure. Therefore, this study aims to develop, provide, and measure the efficacy of a mobile web-based educational program, comparing the emotions of patients undergoing surgery for colorectal cancer treated under the ERAS program to those receiving conventional care.

Among cancer patients, those with higher self-efficacy are known to be better at handling the illness and treatment and enjoy a better quality of life [11]. Depression levels have the largest influence on self-efficacy in self-care in patients with colorectal cancer [12]. Jefford *et al.* [6] showed that, as a reinforcement intervention, self-care was effective at reducing levels of distress and enhancing quality of life.

To improve the effectiveness of treatment in patients with colorectal cancer, it is important for them to actively engage in treatment and nursing to cope with the illness more aggressively. Furthermore, their experience greatly contributes to the planning and provision of nursing [13]. Although some studies have tried to provide more supportive care for cancer survivors and improve the psychosocial effects of care in patients with colorectal cancer [4, 6], scholars have been comparatively less attentive to exploring the effectiveness of interventions addressing the short-term affective aspects of post-surgery discharge under the ERAS program. Some of these concepts have been partially examined in a study investigating post-discharge patients with colorectal cancer who had undergone surgery [1], a study of depression intervention [4], and a study that applied emotional training to caregivers of patients with colorectal cancer [14]. Nonetheless, there has been little comprehensive research examining the affective aspects and quality of life in patients.

Additionally, patients with colorectal cancer undergoing surgery who are treated under the ERAS program require an easily accessible educational program with accurate information. An educational program intended to help patients both during hospitalization and after discharge should be designed to offer this information. Therefore, there is an urgent need for a reliable and easily accessible educational program that is linked to the treatment received at the hospital and can be provided to patients who leave the hospital during the early post-surgery stage. Patients and their caregivers often find it difficult to participate in offline educational programs, which are provided by a few medical staff members for a limited time. To address such limitations, computer technology, auditory and visual media, and supplementary materials, such as leaflets, have been utilized in tandem [15]. Advancements in information technology can be helpful for cancer patients as well, enabling the development of patient-centered educational and intervention programs that cater to the needs of cancer patients by helping them search for information, construct coping strategies, and interact with other patients [16]. The proliferation of smartphones has led to the possibility of further enhancing educational programs for patients with colorectal cancer [17]. Indeed, an increasing number of older patients are using mobile phones with a display and operating system that are equipped with mass memory, allowing communication with other mobile phone-based systems [18].

The current study established a theoretical framework for an educational program for colorectal cancer patients undergoing surgery under the ERAS program. The framework is based on Bandura’s theory of self-efficacy [19] and aims to reduce negative feelings, such as anxiety and depression, while enhancing self-efficacy. Previous studies have examined interventions targeting patients’ affective states. For example, to help patients with colorectal cancer better deal with their illness, such patients were contacted *via* telephone two weeks after discharge, albeit without a structured approach [20]. However, there has been no intervention or mobile-based integrated educational program for patients with colorectal cancer who have been discharged from the hospital after undergoing surgery under the ERAS program. Therefore, this study aims to develop, provide, and measure the efficacy of a mobile web-based educational program, comparing the emotions of patients undergoing surgery for colorectal cancer treated with the ERAS program to those receiving conventional care. We hypothesized that the mobile web-based educational program for these patients would significantly improve their quality of life, self-efficacy, and resilience, and would decrease depression and anxiety.

## 2 MATERIALS AND METHODS

### 2.1 Study Design

A quasi-experimental design and comparative study was conducted among patients with colorectal cancer undergoing surgery treated with the ERAS program at Seoul National University (SNU) hospital in Korea between March 2016 and March 2017. The treatment group received a two-week mobile web-based intervention, whereas the control group only received conventional care. Changes in the primary (quality of life) and secondary outcomes (self-efficacy, anxiety, depression, and resilience) between the treatment and conventional care groups were compared.

### 2.2 Data

#### 2.2.1 Participation Selection

The inclusion criteria were as follows:

1. aged 18 years or older and scheduled to undergo colorectal cancer surgery at SNU hospital;
2. no impairment in mental or cognitive ability;
3. no difficulty reading, speaking, and writing in Korean to communicate with others;
4. able to use a smartphone and other mobile devices; and
5. understood the purpose of this study and provided written informed consent to participate in a mobile web-based educational program.

Exclusion criteria comprised the following:

1. already taking part in other educational programs;
2. unable to communicate in Korean;
3. diagnosed with psychiatric conditions such as depression or with a prior record of cognitive disorders;
4. suffering from a complex illness in addition to colon cancer;
5. expected to undergo surgery again in the near future;
6. developed fever before the surgery;
7. had pneumonia, ileus, or complications around the surgical wound area; or
8. had undergone an emergency surgery.

#### 2.2.2 Data Collection

The quality of life score, the primary endpoint of this study, was utilized to determine the necessary sample size. A two-tailed t-test, which was conducted using the software G*Power (v3.1) with a significance level of 0.05, a group number of 2, an effect size of 0.5, and a power of 0.8, showed that a total of 128 subjects (64 in each group) were needed for this study [21]. Data were collected at the hospital from March 24, 2016 to March 28, 2017. A total of 131 patients with colorectal cancer treated with the ERAS program completed a consent form, but data from only 118 patients were collected, since 13 were excluded due to death, cancelled surgery, or withdrawal of consent (Appendix A). A third party carried out alternate placement by the order of admission registration to assign the patients to the treatment and conventional care groups in a 1:1 ratio. Participants did not know each other or to which group they were allocated. When the treatment group was assigned, they were given a brochure explaining the program and were taught how to participate. The conventional care group, on the other hand, was asked to submit a research consent form and received the conventional, verbal, book-based education. All other interventions were the same across the groups. After completion of a pre-survey by both groups, a two-week educational program was provided upon discharge only to the treatment group. When the program ended, data from both groups were collected by a survey to measure the effects of the program.

### 2.3 Mobile-Based Educational Program

This study developed and offered an educational program to improve the quality of life of patients with colorectal cancer undergoing surgery under the ERAS program by reducing negative feelings, such as anxiety and depression, while enhancing self-efficacy. Some previous studies used a telephone-based program [4] but were not able to verify whether participation in self-care affected anxiety or depression owing to the limited number of telephone calls and time restrictions. Therefore, the mobile web-based educational program was designed to encourage participating patients to play a more active role in managing their illness [22]. This program was designed to enhance self-efficacy by checking actual learning performance on each target item after the patient set his or her own health goals. The completed website was validated by a specialist group that determined its level of relevance for use by analyzing the areas of evaluation, item classification, and each individual item. The validation results for the website attributes of each category achieved a consensus of over 90% and validated eight areas, including the objective, credibility, interaction, update, accessibility, function, design, and security. The completed site was reviewed and modified after being evaluated by the specialist group and tested by the patients.

Based on Bandura’s theory of self-efficacy [19], the program suggested and utilized experiences of accomplishment, vicarious experiences, verbal persuasion, and alleviation of affective arousal as resources for self-efficacy enhancement.

### 2.4 Intervention

#### 2.4.1 Mobile Web-based Educational Group (Treatment Group)

The educational program delivered content through a website accessible *via* mobile devices. Each member of the treatment group was granted an individual ID through which they could join the program. In addition to individual education, feedback, and support from a health professional, participants were encouraged to participate in mobile education *via* short text messages (during the first and second weeks) and telephone counseling (during the second week) three times during the two weeks after discharge. During the initial introduction to the program, the authors provided supplementary materials: manuals that explained how to use the program and a simple booklet that was produced by referring to the learning content index in the program (Appendices B and C). Participation in the education program and learning progress were checked through the website and *via* short text messages three times over the two weeks [23].

#### 2.4.2 Conventional Care Group

Patients who were assigned to the conventional care group were not allowed to take part in the mobile web-based educational program. Instead, they were given booklets and received the conventional education program that consisted of physician- and nurse-led protocol-based individual education and group education with a question and answer session, when applicable. After the experiment concluded, the conventional care group was introduced to the mobile web-based program so that they could use it if they desired.

### 2.5 Assessment

#### 2.5.1 Measurements

Quality of life was measured using the Functional Assessment of Cancer-Colorectal survey (FACIT-C v 4) developed by Ward *et al.* [24]. In addition to the 27 questions in four domains (physical (7), social/family (7), emotional (6), and functional (7)), another nine questions on colorectal cancer were added for a total of 36 questions. Answers were rated on a five-point scale, with higher points indicating a higher quality of life. Cronbach’s α was 0.910 when the tool was first developed, while the Korean version, FACIT-G, has a Cronbach’s α of 0.87. Chen, Goh, Wee, Khoo, and Thumboo [25] examined an Asian population and found the tool had a strong correlation (*r* = 0.85) with Functional Living Index-Cancer (FLIC) scores. Furthermore, previous research by Ward *et al.* [24] observed a high convergent validity between FACIT-C and FLIC (*r* = 0.74). In the current study, Cronbach’s α reliability coefficients for the physical, social/family, emotional, and functional well-being domains were 0.85, 0.83, 0.82, and 0.88, respectively, and that of the FACIT-C subscale was 0.90.

To measure anxiety and depression, this study adopted the Hospital Anxiety Depression Scale, which Zigmond and Snaith [26] developed for people who had visited a general hospital for an illness. The Hospital Anxiety Depression Scale consists of 14 questions: seven questions regarding anxiety and seven questions regarding depression. Each question is rated on a four-point Likert scale, and higher scores indicate a greater sense of anxiety and depression. This tool has a Cronbach’s α of 0.89 for anxiety and 0.86 for depression [27]. In the current study, Cronbach’s α reliability coefficients for the anxiety and depression subscales were 0.87 and 0.82, respectively, and that of the combined anxiety and depression subscales was 0.89.

Self-efficacy was examined using a Korean version of the Hospital Anxiety Depression Scale comprising 17 questions developed by Sherer *et al.* [28] and modified to contain 13 questions. Cronbach's for this tool was 0.886 [29]. In the current study, Cronbach’s α reliability coefficient for the subscales was 0.96.

Finally, resilience was examined using a Korean version of the 25 questions developed by Wagnild and Young [30]. Cronbach's for this tool was 0.886. In the current study, Cronbach’s' α for the tool was 0.961.

#### 2.5.2 Data Analysis

The normality of variables was assessed using the Shapiro-Wilk test. Normally distributed data are reported as the mean ± the standard deviation (*SD*), while non-normally distributed data are reported as the median (range) and categorical data are reported as the number and frequency. To test for differences in demographic characteristics, an independent t-test was performed on normally distributed continuous data and the Wilcoxon rank-sum test was performed on continuous data without a normal distribution. For categorical data, the chi-squared test or Fisher’s exact test was conducted as appropriate. As age, education, employment, and pre-test scores were significantly different between the two groups (*p* < 0.05), an analysis of covariance was used to determine intergroup differences, using these variables as covariates. All statistical analyses were performed using SAS 9.3 (SAS Institute Inc., Cary, NC, USA).

### 2.6 Ethical Considerations

This study was reviewed and approved by the Clinical Research Ethics Committees at both SNU and the SNU hospital. The study was conducted according to the principles of the Declaration of Helsinki. All participating patients were informed of the nature of the study beforehand, and written informed consent was obtained. All participating patients were also informed that their care would not be affected by their decision to participate in this study and that they could withdraw from the study at any time without affecting their care.

## 3 RESULTS

### 3.1 Demographic Characteristics

Of the 139 patients who met the inclusion criteria, eight refused to participate and 13 withdrew. Finally, 118 patients were recruited. The participants in this study had a mean age of 61 years (*SD* = 10) and 61% were men and 39% were women; most participants were married (94.9%) and employed (53.4%). Fifty percent of participants had cancer of Stage 3 or higher, and most did not have an intestinal stoma (95.7%). There were differences between the treatment and conventional care groups in terms of age, education, and employment. Table **1**.

### 3.2 Effects of the Web-based Educational Program

In terms of quality of life, the mean scores of the treatment group were 94.32 (*SD* = 17.71) before receiving the education and 93.69 (*SD* = 12.46) afterwards. In contrast, the conventional care group showed a decline from 87.76 (*SD* = 18.56) before the program to 82.73 (*SD* = 19.57) after four weeks. The analysis of covariance demonstrated a significant gap between the groups in the changes they made after the program (*F* = 10.39, *p* = 0.0017). In particular, the mean scores of the affective status in the treatment group increased from 15.98 (*SD* = 4.3) to 18.31 (*SD* = 3.2) after the mobile web-based educational program was offered, while the conventional care group’s score was 15.29 (*SD* = 4.9) before the program and 15.88 (*SD* = 5.05) four weeks later. The analysis of covariance also demonstrated a markedly significant difference (*F* = 8.18, *p* = 0.0051) in these scores of affective status. Table **2**.

Furthermore, the mobile web-based educational program significantly improved patients’ anxiety, depression, and self-efficacy. More specifically, the treatment group scored 5.54 (*SD* = 4.07) for anxiety before receiving the education and 3.61 (*SD* = 2.48) afterwards. In contrast, the conventional care group showed a decline in anxiety score from 6.24 (*SD* = 4.62) before the program to 6.14 (*SD* = 4.54) after four weeks. The analysis of covariance revealed a significant gap between the groups in the changes demonstrated after the program (*F* = 12.21, *p* = 0.0007). Similarly, the mean depression scores in the treatment group declined from 7.37 (*SD* = 5.06) to 5.85 (*SD* = 3.5) after the mobile web-based educational program was offered, while the conventional care group’s score increased from 8.76 (*SD* = 4.3) before the program to 9.36 (*SD* = 4.7) four weeks later. Analysis of covariance for this metric demonstrated a significant intergroup difference (*F* = 13.66, *p* = 0.0003). The educational program was effective at increasing the treatment group’s self-efficacy, with an increase in mean score from 46.81 (*SD* = 10.29) to 51.34 (*SD* = 8.83), while the conventional care group’s score decreased from 48.8 (*SD* = 9.67) before the program to 47.63 (*SD* = 10.8) four weeks later. The analysis of covariance also demonstrated a significant intergroup difference in this case (*F* = 7.43, *p* = 0.0075). Table **3**.

In addition, the mean scores of the treatment group’s resilience increased from 129.15 (*SD* = 31.46) to 137.19 (*SD* = 29.98) after the mobile web-based educational program was offered, while the scores of the control group showed a moderate rise from 124.92 (*SD* = 31.16) before the program to 125.75 (*SD* = 31.3) four weeks later.

## 4 DISCUSSION

Colorectal cancer is a traumatic and chronic illness that affects an increasing number of individuals due to the world’s aging population and an improved survival rate. Patients with colorectal cancer undergo serious hardship at every stage, including diagnosis, surgery, treatment, and after discharge. As patients with colorectal cancer undergoing surgery with the ERAS program have a shorter hospital stay and less contact with medical professionals, they face various issues, such as the lack of information concerning self-care after leaving the hospital, neglected psychological needs, and insufficient self-efficacy. The resulting distress can lead to other problems, including depression and anxiety [31] and can cause serious problems in daily life activities, such as eating, playing, and sleeping [32]. Therefore, it is essential for patients going through such a stressful course to actively engage in their own treatment, care, and coping to enhance treatment effectiveness.

This study reports the role of a web-based mobile platform in addressing the post-surgical and post-discharge needs of patients with colorectal cancer treated under ERAS. Furthermore, the findings show that the program significantly reduced the negative effects of surgery on the quality of life of those in the treatment group compared to the conventional care group and triggered a sharp improvement in the emotional status of the treatment group. The effect of the program may have been further amplified by the multidimensional system of emotional support involving a tripartite participation of the patient, family members, and medical teams, ensuring mutual and active communication.

This study examined the effect of the platform on patients with colorectal cancer, 50% of whom had cancer at Stage 3 or above and had undergone open surgery under the ERAS program. It is worth noting that, after receiving the mobile web-based educational intervention, the treatment group experienced a positive effect on their quality of life within a short period after hospital treatment. This is further supported by the fact that some patients had a marked improvement in their quality of life within the first month, indicating the necessity of measuring patients’ quality of life during the early post-surgery stages [33]. The quality of recovery scores dropped on postoperative days 1 and 3 but markedly improved from day 6 onward and returned to preoperative levels a month after surgery [34].

In addition, this study demonstrated that the educational program significantly reduced patients’ anxiety and depression and increased their self-efficacy. The significant improvement in self-efficacy and the positive effect on quality of life after participation in the program may be attributable to active engagement in self-care, supporting previous findings [11]. Patients with high self-efficacy are more likely than those with low self-efficacy to engage in health-improving practices and perform physical activities [35]. Moreover, a previous study showed that self-efficacy, quality of life, and psychological well-being are closely linked concepts [36]. Accordingly, the reduction in anxiety and depression and increase in self-efficacy in the treatment group positively affected quality of life.

In the educational program used in this study, patients established their health goals by considering their own disease status. To achieve their goals, patients used a checklist to share their progress in mobile self-learning and understanding at every session and were given rewards by a medical professional.

Medical professionals reviewed the patients’ degree of accomplishment by acquiring information on the need for self-care, the reliability of information provided, and the scope of support, and provided patients with assistance and feedback.

Moreover, the alleviation of affective arousal may be made possible by the vicarious experience of sharing instructional materials and the sharing of disease experiences by patients with colorectal cancer and their families *via* chat rooms in the “colorectal cancer patient community.” In addition, affective arousal can be achieved with education on how to cope with stress and negative feelings. These procedures became feasible using a mobile web-based educational program that the patients could use individually whenever and wherever after discharge. This supports a previous study that found that a mobile-based system can assist cancer patients in more effectively managing their illness by allowing them to interact with medical staff. This approach may also reduce complications by educating patients on how to prevent complications, curb medical costs incurred by long-term hospitalization, and result in better quality of life among patients with colorectal cancer treated under the ERAS program.

These patients have mostly depended on the Internet for information but often find inaccurate information and are left in doubt, leading to feelings of insecurity. Moreover, most caregivers have only focused on the post-surgery and transitional periods between the hospital and the home [37]. Accordingly, an educational program for patients with colorectal cancer undergoing surgery under the ERAS program must be designed to deliver the information that they most need.

With the increasing pressure on the healthcare system, integrating health care services with mobile computing could enhance the emotional well-being and self-efficacy of patients with colorectal cancer by incorporating home care processes into the system [38]. This could improve quality of life, boost productivity, and yield greater treatment efficacy [39]. The use of smartphone applications for patients with colorectal cancer is on the rise, but the potential benefits are offset by the lack of applications developed specifically for patients with colorectal cancer [40].

It is also worth noting that in this study, nurses communicated with patients regarding concerns and addressed health status improvement by checking each patient’s learning performance for each item. Self-efficacy increased through verbal persuasion by nurses.

Although this mobile web-based educational program was verified to be useful and effective for patients, some older patients might feel that it is difficult to work with the mobile equipment. Older patients in the treatment group withdrew more often, and it seemed that they were unfamiliar with and unskilled in the use of mobile web-based programs. As such a possibility may produce a biased result, we attempted to minimize any effect of the differently distributed variables between the two groups by including them as covariates in the analysis. After reviewing all 131 patients assigned equally between the two groups, we found that all variables except education were balanced between the two groups. Consistent with the findings of a previous study, we did not observe any correlation between quality of life and age, education, marital status, or income [41], or any associations between age, sex, education level, and the quality of care [42].

Resources for self-efficacy enhancement, such as those suggested in the current study, can aid patients with colorectal cancer treated under the ERAS program to better deal with negative feelings and to have a greater sense of self-efficacy regarding caregiving. Furthermore, the present study provided the program in a timely manner, thus helping address patient concerns that had been unaddressed due to early discharge. This lowered anxiety and depression and enhanced self-efficacy ultimately lead to a better quality of life among the patients.

## CONCLUSION

In this study, a mobile web-based educational program was developed and used with patients with colorectal cancer who underwent surgery under the ERAS program and had been discharged. Compared to those undergoing the conventional program, patients receiving the educational program experienced a significant decline in anxiety and depression levels and a significant improvement in their self-efficacy, positively impacting their quality of life. The mobile web-based educational program can make education more convenient and reduce complications and expenses incurred from long-term hospitalization and conventional education. In addition, the program can lower distress, anxiety, and depression while enhancing self-efficacy, thereby positively affecting the quality of life of patients treated under the ERAS program.

### Limitations

An important issue for further research is whether the findings and implications of this single-center study can be replicated with other cancers, at other hospitals, or even in other regions. Moreover, the mobile web-based intervention was conducted within two weeks of discharge. Given that some studies indicate that patients complain of anxiety and depression at various times during hospitalization or after discharge, further investigation is required to explore whether there are time-specific differences in program effectiveness. In addition, while our analysis was adjusted for covariance between unequal variables, the possibility of further differences between the groups calls for careful interpretation and generalization of the findings of this study. Further research conducted on a larger sample size and including follow-up to examine the substantive effects of the intervention over a longer time period is recommended.

## Figures and Tables

**Figure:**
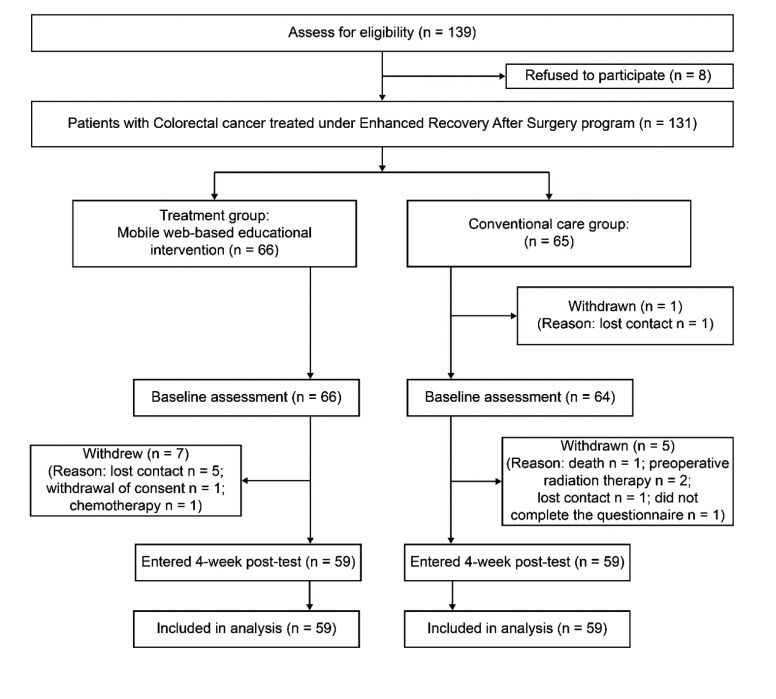


**Table 1:** Participant characteristics (*n* = 118).

**Category**	**Description**	**Treatment Group** **(*n* = 59)**	**Control Group** **(*n* = 59)**	***P*-Value**	**Test Statistics** ^††^
**Age**	Mean ± SD	58 ± 10.13	63 ± 9.77	0.01^†^	– 2.62
**Sex (*n*, %)**	Male	38 (64.41)	34 (57.63)	0.4502^‡^	0.57
	Female	21 (35.59)	25 (42.37)	-	-
**Marriage (*n*, %)**	Yes	57 (96.61)	55 (93.22)	0.6793^§^	0.7024
	No	2 (3.39)	4 (6.78)	-	-
**Education (*n*, %)**	Graduate school or higher	3 (5.17)	2 (3.39)	<0.0001^§^	20.6863
	College	16 (27.59)	10 (16.95)		
	High school	34 (58.62)	24 (40.68)		
	Middle school	3 (5.17)	23 (38.98)		
	Elementary school	2 (3.45)	0 (0)		
**Religion (*n*, %)**	Buddhism	16 (27.12)	14 (23.73)	0.1711^‡^	6.4016
	Protestantism	15 (25.42)	7 (11.86)		
	Catholicism	2 (3.39)	7 (11.86)		
	Others	7 (11.86)	7 (11.86)		
	Nothing	19 (32.2)	24 (40.68)		
**Employed (*n*, %)**	Yes	38 (64.41)	25 (43.1)	0.0208^‡^	5.341
	No	21 (35.59)	33 (56.9)		
**Cancer stage (*n*, %)**	0	2 (3.39)	4 (6.78)	0.2269^‡^	5.6498
	I	14 (23.73)	8 (13.56)		
	II	14 (23.73)	17 (28.81)		
	III	16 (27.12)	23 (38.98)		
	IV	13 (22.03)	7 (11.86)		
**Type of surgery (*n*, %)**	Stoma present	2 (3.45)	3 (5.17)	>0.9999	0.2090
	Stoma not present	56 (96.55)	55 (94.83)		
**Diagnosis (*n*, %)**	Colon cancer	44 (74.58)	33 (55.93)	0.1594^§^	5.6538
	Rectal cancer	13 (22.03)	21 (35.59)		
	Colorectal cancer	2 (3.39)	3 (5.08)		
	Others	0 (0)	2 (3.38)		
**Days of hospitalization**				0.3432^¶^	− 0.94793
	Mean ± SD	7.32 ± 3.23	7.68 ± 3.54		
	Median [range]	6 [4-25]	7 [3-27]		
**Days of hospitalization after surgery**				0.1082^¶^	− 1.60651
	Mean ± SD	4.78 ± 3.14	5 ± 3.12		
	Median [range]	4 [3-23]	4 [2-25]		
**Operative method (*n*, %)**	Right hemicolectomy	13 (22.03)	20 (33.9)	0.2042^§^	4.7348
	Left hemicolectomy	1 (1.69)	3 (5.08)		
	Anterior resection	45 (76.27)	35 (59.32)		
	Hartmann’s operation	0 (0)	1 (1.69)		
**Time after surgery until first drinking water (hours)**				0.4951^¶^	0.68217
	Mean ± SD	18.05 ± 6.63 (*n*=58)	17.78 ± 7.51		
	Median [range]	18 [8-60]	18 [10-61]		
**Use of painkiller in addition to patient-controlled analgesia (*n*, %)**	Yes	24 (40.68)	26 (44.07)	0.709^†^	0.1388
	No	35 (59.32)	33 (55.93)		
**Post-surgery infection (*n*, %)**	Yes	2 (3.57)	3 (5.66)	0.673^§^	0.2715
	No	54 (96.43)	50 (94.34)		

^†^ *p*-value based on t-test ^‡^ *p*-value based on chi-squared test ^§^ *p*-value based on Fisher’s exact test ^¶^ *p*-value based on Wilcoxon rank-sum test ^††^ t-value determined using a t-test, Z using Wilcoxon, chi-squared statistics using chi-squared, and f-value using analysis of covariance. Abbreviation: SD, Standard Deviation.

**Table 2:** Between-group comparisons of the differences in dependent variable scores comprising quality of life (*n* = 118)

**Category**	**Process**	**Description**	**Treatment Group** **(*n* = 59)**	**Control Group** **(*n* = 59)**	***P*-Value^†^**	**Test Statistics^‡^**
**Quality of life**	Baseline	Mean ± SD	94.32 ± 17.71	87.76 ± 18.56	-	-
	Post-test	Mean ± SD	93.69 ± 12.46	82.73 ± 19.57	-	-
	Difference^§^ (post-test minus baseline)	Mean ± SD	− 0.63 ± 17.26	− 5.03 ± 15.78	0.0017	10.39
**Physical status**	Baseline	Mean ± SD	24.27 ± 4.85	23.19 ± 5.21	-	-
	Post-test	Mean ± SD	23.29 ± 3.68	20.59 ± 5.43	-	-
	Difference (post-test minus baseline)	Mean ± SD	− 0.98 ± 4.82	− 2.59 ± 6.01	0.016	5.99
**Family status**	Baseline	Mean ± SD	19.66 ± 4.98	15.9 ± 6.38	-	-
	Post-test	Mean ± SD	19.37 ± 5.06	16.07 ± 6.01	-	-
	Difference (post-test minus baseline)	Mean ± SD	− 0.29 ± 4.1	0.17 ± 5.82	0.1407	2.20
**Affective status**	Baseline	Mean ± SD	15.98 ± 4.3	15.29 ± 4.9	-	-
	Post-test	Mean ± SD	18.31 ± 3.2	15.88 ± 5.05	-	-
	Difference (post-test minus baseline)	Mean ± SD	2.32 ± 4.35	0.59 ± 4.35	0.0051	8.18
**Functional status**	Baseline	Mean ± SD	19.7 ± 5.48	18.08 ± 6.32	-	-
	Post-test	Mean ± SD	17.47 ± 5.86	15.9 ± 7.34	-	-
	Difference (post-test minus baseline)	Mean ± SD	− 2.39 ± 6.25 (*n* = 56)	−2.19 ± 5.5	0.5802	0.31
**Other status**	Baseline	Mean ± SD	16.55 ± 4.09	15.31 ± 4.5	-	-
	Post-test	Mean ± SD	15.25 ± 4.05	14.29 ± 4.2	-	-
	Difference (post-test minus baseline)	Mean ± SD	−1.05 ± 5.19	−1.02 ± 5.54	0.0359	4.52

^†^ *p*-Value by Analysis of Covariance (ANCOVA), with correction for pre-test score, age, education, and employment ^‡^ f-value determined using ANCOVA ^§^ The difference was obtained by subtracting the baseline from post-test values Abbreviation: SD, Standard Deviation.

**Table 3:** Intergroup comparisons of the differences in the dependent variable scores of anxiety, depression, and self-efficacy (*n* = 118).

**Category**	**Process**	**Description**	**Treatment Group (*n* = 59)**	**Control Group (*n* = 59)**	***P*-Value^†^**	**Test Statistics^‡^**
**Anxiety**	Baseline	Mean ± SD	5.54 ± 4.07	6.24 ± 4.62		
	Post-test	Mean ± SD	3.61 ± 2.48	6.14 ± 4.54		
	Difference	Mean ± SD	− 1.93 ± 4	−0.1 ± 3.82	0.0007	12.21
**Depression**	Baseline	Mean ± SD	7.37 ± 5.06	8.76 ± 4.3		
	Post-test	Mean ± SD	5.85 ± 3.5	9.36 ± 4.7		
	Difference	Mean ± SD	– 1.53 ± 6.02	0.59 ± 3.76	0.0003	13.66
**Self-efficacy**	Baseline	Mean ± SD	46.81 ± 10.29	48.8 ± 9.67		
	Post-test	Mean ± SD	51.34 ± 8.83	47.63 ± 10.8		
	Difference	Mean ± SD	4.53 ± 13.28	− 1.17 ± 10.04	0.0075	7.43

^†^ *p*-value by analysis of covariance (ANCOVA), with correction for pre-test score, age, education, and employment ^‡^ f-value determined using ANCOVA. The difference was obtained by subtracting the baseline from post-test values Abbreviation: SD, Standard Deviation.

**Table:** 

**Stage**	**Topic**	**Content**
Baseline (1^st^ survey upon research registration)	Personal information Baseline	-Sex, age, marriage, education, religion, employment, contact information
Development of educational program	Analysis Development System assessment	-Literature review -Focus group interview -Expert interview -Research of currently available colorectal cancer-related websites -Content selection -Display arrangement -Website construction -Composition of smartphone-based educational content -Development of program manuals -Development of evaluation criteria for confirming learning effect -Program simulation and revision ① Expert evaluation ② Evaluation on cancer patient application -Introduction to program, explanation on how to use it, and encouragement for participation *via* individual interview
Application of educational program	Provision of intervention Monitoring	-Setting health goals -Understanding colorectal cancer • What is colorectal cancer? • Diagnosis • Treatment • Prevention -Physical care after discharge • Surgical wound care • Intestinal fistula care • Pain care • Complications care -Emergency management • How to handle an emergency • When to visit the hospital -General management after discharge • Dietary management • Bowel management (diarrhea and constipation) • Daily activity management (exercise, shower, sitz bath, odor care, and sexual life) • Fatigue • Stress management -Community of colorectal cancer patients • Experience of colorectal cancer patients • Experience of caregivers of colorectal cancer patients -Psychological and affective support through listening to experts and feedback -Review of learning content, counseling, and encouragement *via* telephone
Compliance assessment	Participation assessment	-Assignment of ‘task performance table’ by session and verbal persuasion *via* telephone counseling and short text messaging -Assessment on patients’ participation in program and accomplishment of goals
Review (after four weeks)	Post-test	

## LIST OF ABBREVIATIONS

ERAS: = Enhanced Recovery After Surgery
FACIT-C v 4: = Functional Assessment of Cancer-Colorectal tool
FLIC: = Functional Living Index-Cancer
SNU: = Seoul National University

## APPENDICES

### APPENDIX C

http://www.savecolon.or.kr
